# Leptospermum scoparium essential oil is a promising source of mosquito larvicide and its toxicity is enhanced by a biobased emulsifier

**DOI:** 10.1371/journal.pone.0229076

**Published:** 2020-02-20

**Authors:** Ephantus J. Muturi, Gordon W. Selling, Kenneth M. Doll, William T. Hay, Jose L. Ramirez

**Affiliations:** 1 USDA, Agricultural Research Service, NCAUR, Crop Bioprotection Research Unit, Peoria, IL, United States of America; 2 USDA, Agricultural Research Service, NCAUR, Plant Polymer Research Unit, Peoria, IL, United States of America; 3 USDA, Agricultural Research Service, NCAUR, Bio-Oils Research Unit, Peoria, IL, United States of America; 4 USDA, Agricultural Research Service, NCAUR, Mycotoxin Prevention and Applied Microbiology Research Unit, Peoria, IL, United States of America; Al-Azhar University, EGYPT

## Abstract

Synthetic pesticides are the cornerstone of vector-borne disease control, but alternatives are urgently needed to tackle the growing problem of insecticide resistance and concerns over environmental safety. *Leptospermum scoparium* J.R. Forst and G. Forst (manuka) essential oil and its four fractions were analyzed for chemical composition and toxicity against *Aedes aegypti* larvae. The use of bio-based amylose-N-1-hexadecylammonium chloride inclusion complexes (Hex-Am) as an emulsifier for *L*. *scoparium* essential oil was also investigated. Fraction 1 was inactive, fractions 2 (LC_50_ = 12.24 ppm) and 3 (LC_50_ = 20.58 ppm) were more toxic than the whole essential oil (LC_50_ = 47.97 ppm), and fraction 4 (LC_50_ = 35.87 ppm) had similar toxicity as the whole essential oil. Twenty-one chemical constituents were detected in *L*. *scoparium* essential oil compared to 16, 5, 19 and 25 chemical constituents in fractions, 1, 2, 3 and 4 respectively. The two most dominant chemical constituents were calamenene (17.78%) and leptospermone (11.86%) for *L*. *scoparium* essential oil, calamenene (37.73%) and ledene (10.37%) for fraction 1, leptospermone (56.6%) and isoleptospermone (19.73) for fraction 2, cubenol (24.30%) and caryophyllene oxide (12.38%) for fraction 3, and γ-gurjunene (21.62%) and isoleptospermone (7.88%) for fraction 4. Alpha-pinene, ledene, and aromandendrene were 2–7 times less toxic than the whole essential suggesting that the toxicity of *L*. *scoparium* essential oil was either due to other chemical constituents that were not tested or due synergist interactions among chemical constituents. *Leptospermum scoparium* essential oil-Hex-Am emulsion (LC_50_ = 29.62) was more toxic than the whole essential oil. These findings suggest that *L*. *scoparium* essential oil is a promising source of mosquito larvicide and that Hex-Am is an excellent emulsifier for *L*. *scoparium* essential oil for use as a larvicide.

## Introduction

Mosquito-borne diseases remain one of the most pressing public health challenges facing mankind today. Vector control is one of the primary strategies that have effectively been used to break the transmission cycle and has historically relied on the use of synthetic insecticides. Unfortunately, the widespread use of synthetic insecticides has resulted in alarming levels of insecticide resistance among the target mosquito species, raising concerns that current gains in the fight against mosquito-borne diseases could be lost. Moreover, the use of some synthetic insecticides in vector control has been discontinued or restricted due to their potential to disrupt ecological processes and cause harm to non-target organisms. These challenges have reinforced the urgent need for effective and environmentally-friendly vector control strategies.

Some plants produce essential oils that contain a spectrum of chemical compounds which provide protective role against herbivores and pathogens. Some of these essential oils have insecticidal and repellent activity against mosquitoes [1–6] and possess other traits that make them suitable alternatives to synthetic insecticides. These include low mammalian toxicity, rapid degradation in the environment, limited non-target effects, multiple modes of action that may inhibit development of insecticide resistance, and commercial availability at an affordable cost [7, 8]. As a result, significant research effort has been devoted towards the discovery and development of essential oil-based insecticides for pest and vector management.

Manuka (Myrtaceae: *Leptospermum scoparium* J.R. Forst and G. Forst) also known as “tea tree”, is one of the most abundant and widespread indigenous shrub species in New Zealand. Early records report the traditional use of the bark, leaves, sap, and seed capsules from manuka for treatment of various diseases and ailments including fever, cough, mouth and throat sores, running nose, dysentery, diarrhea, colic pain, breast inflammation, back stiffness, eye problems, and scald and burn injuries [9, 10]. Essential oil derived from *L*. *scoparium* is also used as a strong antimicrobial and antifungal agent in creams, soaps, toothpastes and other preparations [11, 12]. During World War II, *L*. *scoparium* essential oil was provided in the first aid kits of serving Australian soldiers for use as a general antimicrobial agent and insect repellent [10]. Other studies have demonstrated the toxicity of *L*. *scoparium* essential oil and some of its fractions against arthropods of economic and medical significance including the spotted wing drosophila *Drosophila suzukii* Matsumura, itch mite, *Sarcoptes scabiei* Linnaeus, poultry red mite, *Dermanyssus gallinae* De Geer, stored food mite, *Tyrophagus putrescentiae* Schrank, and house dust mites, *Dermatophagoides farinae* Hughes and *D*. *pteronyssinus* Troussart [13–16]. *Leptospermum scoparium* essential oil has also been shown to be an attractive bait for the redbay ambrosia beetle, *Xyleborus glabratus* Eichhoff [17]. A recent study by our research group also demonstrated that *L*. *scoparium* essential oil is toxic to *Aedes aegypti* Linnaeus larvae (LC_50_ = 53.0 ppm) and interacted synergistically with oregano essential oil and antagonistically with clove bud essential oil [4]. In general, however, knowledge regarding the insecticidal activity of *L*. *scoparium* essential oil against arthropods of medical and economic significance is limited. Additionally, most studies on insecticidal properties of essential oils focus on the whole essential oil, yet some studies have shown that bioassay-guided fractionation of some essential oils may yield fractions that are more toxic than the oil itself [3, 18–20]. The use of essential oils as mosquito larvicides also remains a challenge due to their chemical instability, high volatility, and poor solubility in water. Thus, technologies that improve the solubility and environmental stability of essential oils when used as biopesticides are urgently needed.

Oil-in-water emulsions are considered to be efficient delivery systems for hydrophobic compounds by dispersing the lipid phase as a colloidal dispersion [21]. Here, two immiscible liquids are stabilized through the addition of a surfactant (emulsifier) which prevents droplet coalescence by lowering the interfacial tension [22]. Amylose from starch has attracted significant interest as a low-cost material for the synthesis of emulsifying agents. When amylose is combined with suitable ligands such as fatty amine salts, the hydrophobic portion of the ligand associates with the hydrophobic internal cavity of the amylose helix to form water-soluble amylose-inclusion complexes [23, 24]. These complexes have been shown to be surface active polymers with superior emulsion activity compared to commercial modified starch emulsifiers [25] and have been used to reduce volatility, increase stability, enhance bioactivity and extend the shelf life of the target bioactive compounds [25, 26]. Additionally, amylose inclusion complexes are biodegradable and non-toxic, making them appealing emulsifiers for the development of ecofriendly biopesticides [27].

In this study we analyzed the chemical composition of *L*. *scoparium* essential oil and its fractions and evaluated their toxicity against larvae of the yellow fever mosquito, *Aedes aegypti*. We also evaluated the use of amylose-N-1-hexadecylammonium chloride inclusion complexes (Hex-Am) as emulsifiers for *L*. *scoparium* essential oil for use as mosquito larvicides. Our overall goal was to develop a better understanding of the insecticidal activity of *L*. *scoparium* essential oil against disease vectors and to generate new knowledge that may guide the development of effective biopesticides based on essential oils.

## Materials and methods

### Preparative chromatography

Separation of *L*. *scoparium* essential oil fractions was performed using preparative flash chromatography (Cheetah MP200, Bonna-Agela Technologies Inc., Newcastle, DE). The column (Supel Dlash Catridge, 80g, 40–60 μm silica) was equilibrated with hexane for 10 min at a flow rate of 60 mL per min. The oil sample (5 mL) was injected into the column using a 10-mL syringe and the column was developed with hexane-ethyl ether gradient method over 27 minutes as follows: 100% hexane for 2 min, 0–100% ethyl ether for 20 min, and 100% ethyl ether for 5 min. The effluent was monitored at 254 nm and fractions were collected by volume (60 mL). Fractions containing each absorbance peak were pooled and placed in the fume hood for evaporation of organic solvent. The procedure was repeated until adequate amounts of different fractions were obtained. All fractions were labeled and stored in amber-colored glass bottles until use.

### Gas Chromatography-Mass Spectrophotometry (GC-MS) analysis

Identification of the chemical constituents of *L*. *scoparium* essential oil and its fractions was accomplished as previously described [4]. Briefly, two different Agilent 7890 (Santa Clara, CA) gas chromatographs, each using Agilent’s Mass Hunter software were used to acquire and process the data. A 5975 mass spectrometry detector using NIST05 library (National Institute of Standards and Technology, Gaithersburg, MD) was employed for product identification, whereas flame ionization detection (FID) was used for quantitation. Samples of ~10 μL were diluted in 1 mL of heptane and 1 μL was injected by autosampler using a 50:1 split ratio and analyzed on Agilent/J&W DB35-MS column (30m × 320 mm, 0.25 mm film thickness). Helium flow in the column was maintained at 1.37 mL per minute. Oven temperature was programmed at 40°C for 3 min, 10°C min^-1^ to 190°C for 5 min, and 25°C min^-1^ to 340°C. Commercial compounds were purchased when available, diluted in heptane, and used for comparison of retention time. To determine the relative retention time, a GC sample of alkanes, ranging in size from decane to tetracosane (10 to 24 carbon atoms) was made and ran on the GC and GC-MS under identical conditions to the sample analysis. The retention times of each of the alkanes was determined, and then all of the relative retention times were calculated according to the formula 7 in ASTM D6730-19 as follows:

$$RRI=100\;x\;(n+\left(\left(log\;T_{sample}‐log\;T_{n}\right)/\left(log\;T_{n+1}–log\;T_{n}\right)\right)\;where\;n\;is\;the\;number\;of\;carbons\;in\;the\;preceding\;paraffin.$$

### Preparation of amylose-complexes and oil emulsions

Amylose-complexes were produced following the procedure outlined previously [28–30]. A dispersion of high-amylose starch (100.0 g of starch) and deionized water (1800 mL) was passed through a Penick & Ford laboratory model continuous steam jet cooker (Penford Corp., Cedar Rapids, IA) operating under the following conditions: hydroheater temperature 140°C, steam back pressure 380 kPa, steam line pressure 550kPa and pumping rate of 1 L min^−1^. Cooked dispersions were collected in a Dewar flask to prevent rapid temperature loss. A solution of N-1-hexadecylammonium chloride was prepared as previously described [29] and added to the hot starch dispersion immediately after jet-cooking. The mixture was rapidly stirred for 1 min, and then cooled to 25°C in an ice bath. Spray drying of amylose-N-1-hexadecylammonium chloride inclusion complexes (Hex-Am) was performed using a Niro atomizer spray dryer (Niro, Columbia, MD, USA) as previously described [29]. Materials were collected and stored at room temperature until use. Spray dried amylose-complexes were used as emulsifiers to prepare oil-in-water (O/W) emulsions. Emulsions were prepared by mixing water, *L*. *scoparium* essential oil, and spray-dried Hex-Am at 92.5: 5: 2.5 ratio, respectively. A mixture totaling 10 g, was placed in a 30 mL glass beaker and homogenized for 180 seconds at 20,000 rpm using a Power Gen 35 handheld micro homogenizer (Fisher Scientific, Pittsburgh, PA).

### Dynamic light scattering

Dynamic light scattering (DLS) analysis to determine the particle size and distribution was conducted using a Horiba LB-550 Dynamic Light Scattering Particle-Size Analyzer (HORIBA Instruments Incorporated, Irvine, CA). The analysis was conducted at 25°C using a 1 cm path-length cell having a volume of 1.25 mL. Aqueous emulsions (minimum three samples tested) of *L*. *scoparium* essential oil and Hex-Am were diluted ~1000x to obtain spectra. Horiba software was used to analyze and process the hydrodynamic diameter distribution data to determine the median hydrodynamic diameter. Intensity % for each diameter was calculated by dividing its value by the total area for the spectral curve multiplied by 100.

### Larvicidal bioassays

*Aedes aegypti* (Rockefeller strain) larvae were reared on yeast: lactose albumin (1:1) diet in batches of ~200 larvae at 26°C, 70% relative humidity (RH) and 10:14 h (light: dark cycle). Larvae from all rearing containers were pooled before the bioassays. With exception of the water volume and the starting number of larvae per container, the toxicity bioassays followed the standard World Health Organization guidelines [31]. Twenty late third instar larvae of *Ae*. *aegypti* were added into 120 mL of DI water held in 400 mL tripour beakers. Treatments included *L*. *scoparium* essential oil purchased from Sigma-Aldrich and its four fractions obtained via flash chromatography. The oil and its fractions were diluted in absolute ethanol to create stock solutions of similar concentrations to oil emulsions (50,000 ppm). The treatments were tested at varying concentrations depending on their degree of toxicity. *Leptospermum scoparium* essential oil, fraction 1 and fraction 4 were tested at 7 concentrations ranging from 20–80 ppm. Fraction 2, fraction 3 and *L*. *scoparium* essential oil-Hex-Am emulsion were more toxic and were tested at lower concentrations. Fractions 2 and 3 were tested at 7 concentrations ranging from 5–35 ppm for fraction 2 and 16–34 ppm for fraction 3. *Leptospermum scoparium* essential oil-Hex-Am emulsion was tested at 6 concentrations ranging from 20–45 ppm. A control group was treated with absolute ethanol without oil/fraction/emulsion treatment. Each treatment was replicated 3 times, and 3 separate trials with different batches of mosquitoes were conducted. The containers were held at room temperature and the total number of larvae surviving 24 hours post-treatment were counted and recorded. Probit analysis conducted using “ecotox” package in R version 3.3.2 was used to calculate the LC_50_ and LC_90_ values for each oil/fraction/emulsion. To determine the contribution of some individual constituents to the toxicity of *L*. *scoparium* essential oil, three *L*. *scoparium* essential oil chemical constituents that were commercially available at affordable prices were purchased from Millipore Sigma (Saint Louis, MO) and tested at a concentration of *L*. *scoparium* essential oil (70 ppm) expected to kill 90% of the test larvae. The chemical constituents were alpha-pinene, ledene, and aromandendrene and were tested using the experimental procedures described above.

## Results

**Fig 1:**
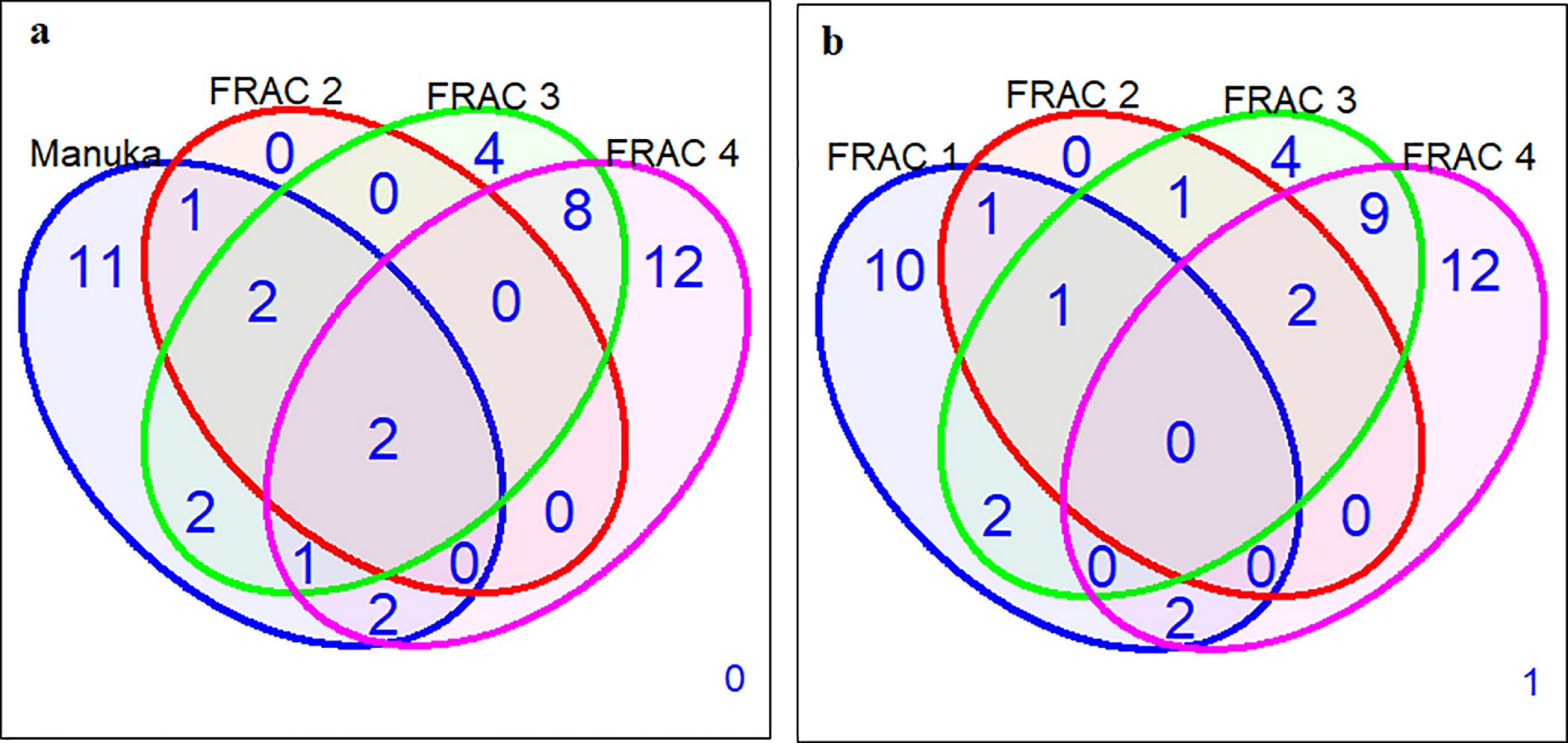
Venn diagram summarizing the overlap of chemical constituents between manuka (*L*. *scoparium*) essential oil and its fractions.

A total of 29.5 g of *L*. *scoparium* essential oil was processed yielding 13.06 (44.27%), 7.05 (23.90%), 1.43 (4.85%) and 0.96 g (3.25%) of fractions 1, 2, 3, and 4 respectively. GC-MS analysis revealed qualitative and quantitative differences in the chemical composition of *L*. *scoparium* essential oil and its fractions (Table 1). Twenty-one chemical constituents were detected in *L*. *scoparium* essential oil compared to 16, 5, 19 and 25 chemical constituents in fractions, 1, 2, 3 and 4 respectively (Table 1). Calamenene (17.78%), leptospermone (11.86%), α-selinene (7.17%) and α-cadinene (6.40%) were the four most abundant chemical constituents in *L*. *scoparium* essential oil. The four most abundant chemical constituents in fraction 1 were calamenene (37.73%), ledene (10.37%), α-selinene (9.20%), and α-copaene (7.96%). Fraction 2 was predominantly leptospermone (56.6%), isoleptospermone (19.73%), flavesone (16.82%), and γ-muurolene (5.42%). For fraction 3, the dominant constituents were cubenol (24.30%), caryophyllene oxide (12.38%), leptospermone (10.89%), and flavesone (6.78%). The dominant constituents in fraction 4 were γ-gurjunene (21.62%), isoletospermone (7.88%), eudesma-4(14),11 diene (6.61%), and unidentified compound (6.00%). Venn diagrams were used to summarize the chemical constituents that were present/absent in *L*. *scoparium* essential oil and its fractions (Fig 1). All 16 constituents detected in fraction 1 were present in the whole essential oil, but 5 constituents present in the whole essential oil were not detected in fraction 1. These were α-pinene, isoleptospermone, leptospermone, cubenol, and γ-muurolene. Similarly, all 5 chemical constituents detected in fraction 2 were present in the whole essential oil. With exception of α-cubebene, these compounds were more abundant in fraction 2 than in the whole essential oil. Seven compounds were shared between *L*. *scoparium* essential oil and fraction 3, 14 were only detected in *L*. *scoparium* essential oil and 12 were only detected in fraction 3. Five constituents were shared between *L*. *scoparium* essential oil and fraction 4, with 16 constituents only detected in *L*. *scoparium* essential oil and 20 constituents only detected in fraction 4. Overall, 11, 0, 4, and 12 compounds were unique to *L*. *scoparium* essential oil, fraction 2, fraction 3 and fraction 4, respectively and only 2 constituents were shared among the four treatments. When only the four fractions were considered, 10, 0, 4, and 12 constituents were unique to fractions 1, 2, 3 and 4 respectively and no compounds were shared among all four fractions.

**Table 1:** Chemical composition of *L*. *scoparium* essential oil and its fractions. LP, *L*. *scoparium* essential oil, F1-F4, fractions 1–4. Also included are retention time from the GC-FID with relative retention index (RRI), and the major fragmentations ions observed by GC-MS listed in order of relative abundance. Dash (-) indicates that the compound was not detected.

Compound	RT (min)	RRI	M/z	LP	F1	F2	F3	F4
α-Pinene	7.66	<1000	108, 64, 117, 116, 109	1.98	-	-	-	-
α-Copaene	14.37	1268	161, 105, 119, 91, 93	4.17	5.65	-	-	-
β-Copaene	14.86	1279	119, 161, 105, 93, 91	5.88	7.96	-	-	-
β-Elemene	15.16	1286	93, 81, 67, 107, 68	1.01	1.53	-	-	-
α-Gurjunene	15.34	1290	204, 105, 161, 189, 119	1.19	1.43	-	-	-
(E)-Caryophyllene	15.66	1297	93, 133, 91, 79, 69	3.10	3.38	-	-	-
Aromandendrene	15.89	1506	91, 161. 105, 93, 107	2.72	4.02	-	-	-
α-Cubebene	16.11	1525	161, 105, 119, 204, 91	6.06	0.77	1.36	-	-
Zonarene	16.35	1545	161, 105, 204, 119, 91	4.48	2.40	-	-	-
α-Amorphene	16.44	1553	161, 105, 119, 91, 93	1.30	1.89	-	1.48	-
Ledene	16.70	1574	93, 105, 107, 121, 67	5.42	10.37	-	-	-
α-Selinene	16.76	1579	189, 105, 161, 133, 204	7.17	9.20	-	-	1.69
δ-Cadinene	17.03	1601	161, 119, 204, 134, 105	6.40	7.85	-	-	
β-Selinene	17.12	1609	161, 204, 81, 119, 105	1.74	1.18	-	-	1.30
Cadina-1,4-diene	17.27	1622	119, 105, 161, 91, 204	5.87	3.64	-	-	-
Calamenene	17.35	1630	159, 129, 128, 202, 131	17.78	37.73	-	2.78	-
β-Bisabolene	17.54	1646	157, 143, 200, 142, 141	-		-	2.45	-
Flavesone	17.61	1652	252, 139, 182, 237, 96	4.54	0.99	16.82	6.78	-
Alloaromandendrene	17.80	1669	109, 161, 82, 105, 93	-	-	-	2.76	-
Viridiflorol	18.07	1692	121, 81, 108, 222, 93	-	-	-	5.19	2.26
γ-Gurjunene	18.25	1707	91, 159, 205, 105, 119	-	-	-	-	21.62
Spathulenol	18.31	1711	205, 91, 119, 159, 105	-	-	-	2.52	1.83
Caryophyllene oxide	18.35	1714	79, 93, 91, 69, 95	-	-	-	12.38	5.09
Isoleptospermone	18.47	1724	266, 251, 196, 96, 178	4.40	-	19.73	1.60	7.88
Leptospermone	18.64	1736	196, 266, 251, 96, 69	11.86	-	56.60	10.89	-
Unknown	18.70	1741	149, 59, 107, 164, 135	-	-	-	4.77	2.78
Cubenol	18.77	1746	119, 161, 105, 204, 91	1.23	-	-	24.30	1.34
Unknown	18.87	1753	189, 161, 204, 91, 105	-	-	-	-	1.76
γ-Muurolene	18.91	1756	161, 119, 179, 105, 204	1.69	-	5.49	5.42	3.29
Unknown	19.01	1764	161, 204, 105, 119, 162	-	-	-	1.56	2.33
Unknown	19.08	1769	121, 105, 161, 91, 93	-	-	-	-	6.00
Unknown	19.16	1775	161, 189, 119, 105, 95	-	-	-	-	4.64
γ-Eudesmol	19.31	1786	161, 149, 204, 189, 95	-	-	-	4.11	5.71
β-Eudesmol	19.37	1790	59, 149, 164, 109, 108	-	-	-	-	3.62
Isoaromadendrene epoxide	19.43	1794	91, 93, 79, 105, 107	-	-	-	-	2.92
Eudesma-4(14),11 diene	19.50	1799	81, 135, 189, 204, 93	-	-	-	-	6.61
Unknown	19.59	1802	159, 132, 135, 91, 107	-	-	-	2.01	-
Unknown	19.69	1805	175, 157, 143, 142, 126	-	-	-	3.52	3.82
Unknown	19.75	1807	159, 91, 117, 118, 105	-	-	-	-	1.01
Unknown	19.89	1811	159,91, 132, 105, 93	-	-	-	-	1.18
Unknown	19.94	1813	164, 206, 122, 121, 91	-	-	-	-	4.78
Unknown	20.77	1836	Unclear	-	-	-	-	2.10
Unknown	21.52	1856	Unclear	-	-	-	-	4.45
Calamenol 1	22.68	1887	175, 176, 160, 145, 218	-	-	-	1.90	3.82
Unknown	26.83	2099	91, 244, 243, 314, 296	-	-	-	3.57	-

**Fig 2:**
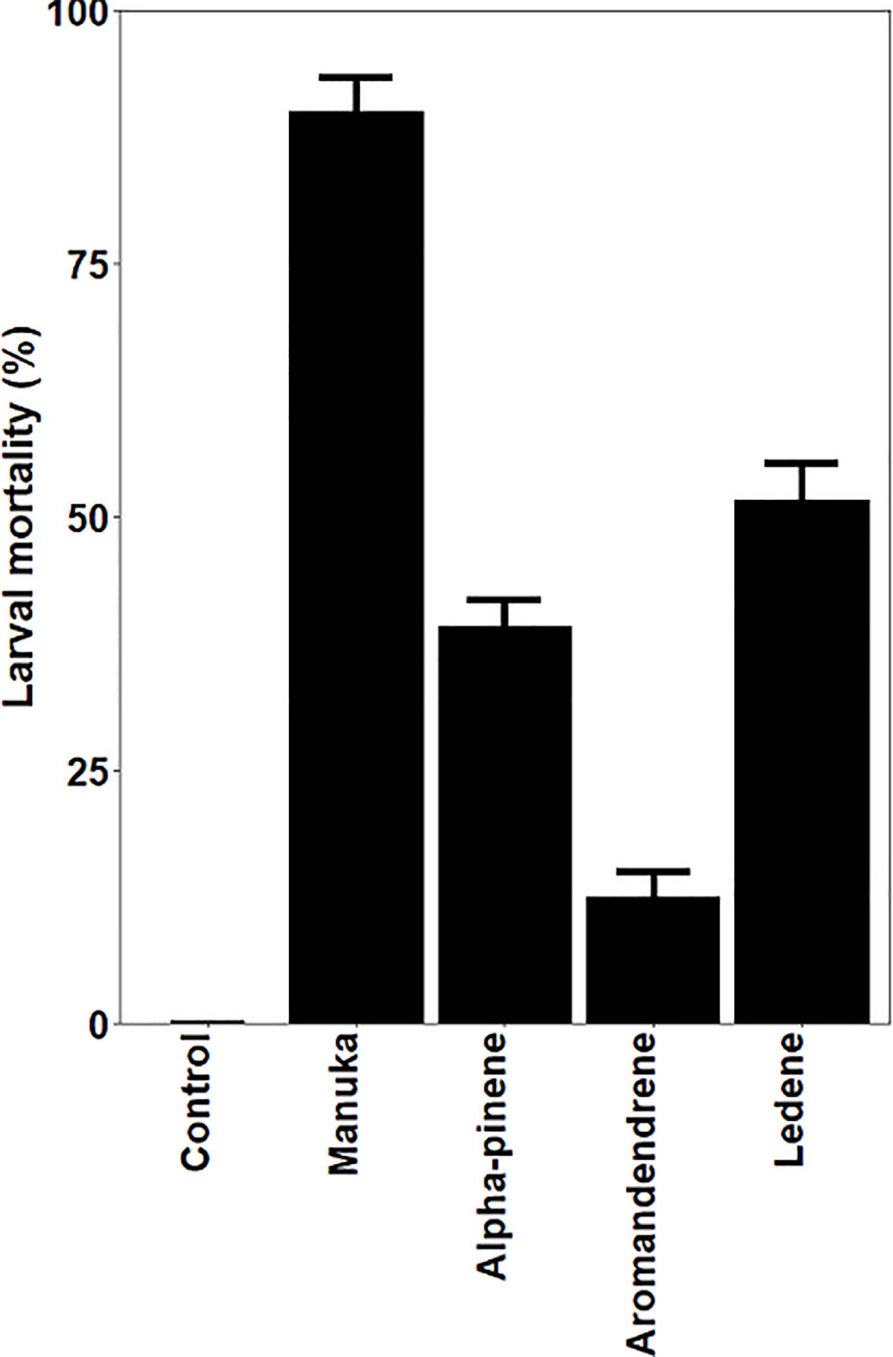
Toxicity of three chemical constituents of manuka (*L*. *scoparium*) essential oil against *Aedes aegypti* larvae relative to whole essential oil. Error bars represent the standard error of the mean.

The larvicidal activity of *L*. *scoparium* essential oil fractions and emulsions against *Ae*. *aegypti* was evaluated relative to the whole essential oil. Fraction 1 was inactive, fractions 2 (LC_50_ = 12.24 ppm) and 3 (LC_50_ = 20.58 ppm) were 4 and 2 times more toxic than the whole essential oil (LC_50_ = 47.97 ppm), and fraction 4 (LC_50_ = 35.87 ppm) had similar activity as the whole essential oil (Table 2). The three chemical constituents of *L*. *scoparium* essential oil tested (α-pinene, ledene, and aromandendrene) were 2–7 times less toxic than the whole essential oil (Fig 2). *Leptospermum scoparium* essential oil-Hex-Am emulsion (LC_50_ = 29.62 ppm) was more toxic to *Ae*. *aegypti* larvae than the whole essential oil. Dynamic light scattering analysis of *L*. *scoparium* essential oil-Hex-Am emulsion revealed that the complexes had a median (± SD) hydrodynamic diameter of 1.96 ± 0.74 microns.

**Table 2:** LC_50_ and LC_90_ values for *Leptospermum scoparium* essential oil and its fractions and emulsions produced with hexadecyl ammonium chloride amylose inclusion complexes. ND, not determined because it was outside the range of concentrations tested. LP, *Leptospermum scoparium*.

Treatment	LC_50_ (95% CI)	LC_90_ (95% CI)	Slope
LP	47.97 (45.72–50.22)	66.62 (62.48–72.69)	y = 8.99x - 15.10
Fraction 1	ND	ND	ND
Fraction 2	12.24 (10.94–13.43)	18.78 (16.94–21.70)	y = 6.90x - 7.50
Fraction 3	20.58 (19.85–21.27)	26.07 (25.01–27.49)	y = 12.49x - 16.41
Fraction 4	35.87 (33.09–38.49)	79.31 (71.50–90.78)	y = 3.72x - 5.78
Hex-Am	29.62 (28.97–30.25)	35.92 (34.94–37.13)	y = 15.29x -22.51

## Discussion

*Leptospermum scoparium* essential oil is known for its many medicinal applications, but its insecticidal properties remain poorly understood. Here, we show that *L*. *scoparium* essential oil can serve as an important source of larvicides for mosquito control. Fractions 2 and 3, respectively were 4 and 2 times more toxic than the whole essential oil, while fraction 4 had similar toxicity as the whole essential oil. The World Health Organization (WHO) has not established a standard criterion for determining the larvicidal activity of natural products but several scientists have developed their own criteria. Komalamisra et al. [32] considered products showing LC_50_ <50 mg/L active, 50 mg/L<LC_50_<100 mg/L moderately active, 100 mg/L<LC_50_<750 mg/L effective, and LC_50_>750 mg/L inactive. Kiran et al. [33] considered compounds with LC_50_<100 mg/L to exhibit a significant larvicidal effect. Cheng et al. [34] considered compounds with LC_50_<50 mg/L highly active, 50 mg/L<LC_50_<100 mg/L active, and LC_50_>100 mg/L inactive. Based on these criteria, *L*. *scoparium* essential oil and three of its four fractions can be considered active/highly active. Moreover, the use of Hex-Am emulsifier enhanced the toxicity of *L*. *scoparium* essential oil against the mosquito larvae. These findings highlight the need to explore the potential for development and commercialization of *L*. *scoparium* essential oil as a mosquito larvicide.

Most of the chemical constituents identified in this study have been reported in *L*. *scoparium* essential oil albeit at different quantities [9, 13, 16, 35, 36]. These differences are expected because the chemical composition of essential oils is known to vary by plant geographic origin, stage of development, growing conditions, developmental stage of the plant, method of extraction, solvent used for extraction, and photosensitivity of some compounds in the extract [36–38].

*Leptospermum scoparium* essential oil has been shown to be toxic against the spotted wing drosophila *Drosophila suzukii* Matsumura [16] and several species of mites [13–16], and also can synergize the activity of some essential oils against mosquito larvae [4]. *Leptospermum scoparium* essential oil has also been shown to be an attractive bait for the redbay ambrosia beetle, *Xyleborus glabratus* [17]. The finding that some *L*. *scoparium* essential oil fractions are more toxic than the whole essential oil is similar to our recent findings with the Italian honeysuckle (*Lonicera caprifolium* Linnaeus) essential oil where the whole essential oil (LC_50_ of 34.4 mg/L) was 2 times less toxic to *Ae*. *aegypti* larvae than 4 of its 5 fractions (LC_50s_ = 20.6, 19.7, 18.6, and 17.7 mg/L for fraction B, C,D and E, respectively) [3]. Similar findings were also reported on parsley (*Petroselinum crispum* Mill.(Fuss)) essential oil where the LC_50s_ for fractions 1, 3, and 4 against *Ae*. *aegypti* larvae were 0.49, 0.88 and 0.01 mg/L respectively, compared to 4.19 mg/L for the whole essential oil [20]. Sweet orange, *Citrus sinensis* (L.) Osbeck essential oil was also a less potent fumigant against the red imported fire ant *Solenopsis invicta* Buren compared to its fractions [19]. We were unable to identify the chemical constituents responsible for the larvicidal properties of *L*. *scoparium* essential oil since the three chemical constituents tested (α-pinene, ledene, and aromandendrene) were less toxic than the whole essential oil, and our efforts to test additional chemical constituents did not materialize because the other chemical constituents of *L*. *scoparium* essential oil were either too expensive or not commercially available. Thus, it is possible that the toxicity of *L*. *scoparium* essential oil is due to one or more chemical constituents that were not tested, or due to synergistic interactions between multiple chemical constituents. Further studies are needed to clarify this.

The two fractions that were more toxic than the whole essential oil (fractions 2 and 3) constituted 28.75% of the total essential oil processed, with fraction 2 accounting for 23.9% of the total oil. Because *L*. *scoparium* essential oil is generally recognized as safe to humans and environment and is commercially available in large quantities and affordable cost, our findings suggest that commercial development and application of *L*. *scoparium* essential oil as a mosquito larvicide is feasible. However, it is also important to note that fraction 1 which was inactive, accounted for 44.27% of the total yield suggesting that large scale production of fractions 2 and 3 would also yield large amounts of fraction 1. This fraction would be useless with regard to mosquito control and might pose substantial disposal challenges. Further studies should be conducted to identify the value-added uses of fraction 1 in order to improve the efficiency and economic viability of this process. These studies may include bioassays with other insects of medical, veterinary and wildlife significance, and tests for antimicrobial activity and potential application in cosmetics and pharmaceutical industries.

We did not investigate the mechanism(s) underlying the enhanced toxicity of *L*. *scoparium* essential oil fractions relative to whole essential. However, previous studies have shown that different chemical constituents present in essential oils or their fractions may act in synergy through enhanced penetration, targeting multiple sites, and exhibiting multiple modes of action [39–41]. There also are reports that when exposed to a mixture of terpenes, the insect may preferentially oxidize the major terpene in the mixture while the minor terpene acts as a toxicant with higher toxicity than when used alone [42]. We observed qualitative and quantitative differences in the chemical compositions of *L*. *scoparium* essential oil and its fractions and it is likely that at least one of the mechanisms described above may have contributed to the enhanced toxicity of some oil fractions relative to the whole essential oil. For example, the major compounds detected in fraction 1 which was inactive, were either absent or found in much lower quantities in other fractions (e.g. calamenene, ledene, α-selinene, α-copaena, δ-cadinene, β-copaena, aromandendrene). The five compounds detected in fraction 2 were also detected in substantial amounts in some of the other active fractions as well as in the whole essential oil suggesting their potential contribution to the observed bioactivity. In addition, a good number of chemical constituents that were present in *L*. *scoparium* essential oil fractions especially fractions 3 and 4 were not detected in the whole essential oil and vice versa. A simple explanation for the presence of some constituents in the fractions but not in the whole essential oil would be that these chemicals were present in undetectable amounts in the whole essential oil but became enriched in the fractions when some major compounds were either removed or their abundance reduced through fractionation. For example, γ-Gurjunene accounted for 21.6% of fraction 4 but was neither detected in the other fractions nor in the whole essential oil. The 29.5 g of oil that was processed yielded only 0.96 g of fraction 4. This is a concentration factor of 31-fold. Thus, a component of 21.6% in fraction 4 would comprise only 0.7% of the original oil, which is below our detection limits. Under this scenario, the enhanced toxicity of *L*. *scoparium* essential oil fractions may have resulted from enrichment of some of bioactive compounds and reduction in the concentration of some inactive compounds. Essential oils also tend to be highly volatile, thermally unstable, and quite sensitive to oxidation [27, 43]. Therefore, the loss of some components may have resulted from vaporization and/or chemical degradation during fractionation.

Enhancing the water dispersibility of essential oils is another effective method for improving insecticidal activity of essential oils. Our results show that amylose-N-1-hexadecylammonium chloride inclusion complexes can be used as an emulsifier to improve the solubility and efficacy of *L*. *scoparium* essential oil in aqueous systems. The higher toxicity of emulsions relative to the whole essential oil may be due to the reduction in droplet sizes which may have improved their effective distribution in the water column and interaction with insect tissues [44]. Amylose inclusion complexes have been shown to be surface active agents that reduce the interfacial tension at the oil-water interface and inhibit flocculation and coalescence of oil droplets [25]. Additionally, high molecular weight polymers such as the amylose complexes can substantially inhibit emulsion breakdown via Ostwald ripening by forming a thick, high elastic modulus polymer coating around the oil droplets [25, 45].

The ligands bound in the Hex-Am amylose complexes are cationic fatty ammonium salts with 16 carbon alkyl tails [46]. The amylose complexes may be forming highly stable Pickering emulsions with the *L*. *scoparium* essential oil; where the polymer particles adhere to the oil droplets forming a steric barrier on the surface [25, 47]. Pickering emulsions using garlic and asafoetida essential oils with Hex-Am are highly resistant to destabilization processes and are suitable for long term (6 months) storage [48]. For commercial application however, studies covering a longer time frame are needed to fully elucidate the stability of amylose inclusion complex emulsions.

This study focused on the lethal effects caused by *L*. *scoparium* essential oil, its fractions and emulsions. However, essential oils are also known to cause a variety of sublethal effects that are detrimental to insect survival and reproduction. These effects include repellency, irritability, altered respiratory activity, changes in swimming pattern, and reduced adult emergence, longevity, fertility, fecundity and natality [49–52]. Future studies evaluating both the lethal and sublethal effects of *L*. *scoparium* essential oil, its fractions and emulsions could reveal the full spectrum of their biological effects against mosquitoes. These studies should be conducted under a range of temperatures because the insecticidal activity of some essential oils and their constituents is influenced by post-application temperature [53].

In summary, our results show that *L*. *scoparium* essential oil and three of its four fractions examined are toxic to mosquito larvae and could be harnessed as a source of bio-based mosquito larvicides. In addition, we show that amylose-N-1-hexadecylammonium chloride inclusion complexes are a promising emulsifier and increases the toxicity of *L*. *scoparium* essential oil in aqueous dispersions. Amylose inclusion complexes can be composed of bio-based materials that are relatively safe and are made from low cost materials and processes [25]. Their use as emulsifiers for essential oil biopesticides is therefore appealing both in terms of cost and environmental and public health safety. Further studies are needed on the effects of *L*. *scoparium* essential oil and its fractions on non-target organisms, and the potential development and commercialization of amylose inclusion complexes as emulsifiers for essential oil-based insecticides for mosquito control.
